# Junctional Adhesion Molecule-C Mediates the Recruitment of Embryonic-Endothelial Progenitor Cells to the Perivascular Niche during Tumor Angiogenesis

**DOI:** 10.3390/ijms21041209

**Published:** 2020-02-11

**Authors:** Marcus Czabanka, Lucia Lisa Petrilli, Susanne Elvers-Hornung, Karen Bieback, Beat Albert Imhof, Peter Vajkoczy, Maria Vinci

**Affiliations:** 1 Department of Neurosurgery, Universitätsmedizin Charitè, 10117 Berlin, Germany; marcus.czabanka@charite.de; 2 Department of Neurosurgery Medical Faculty of the University of Heidelberg, 68167 Mannheim, Germany; maria.vinci@opbg.net; 3 Department of Onco-haematology, Cell and Gene Therapy, Bambino Gesù Children’s Hospital – IRCCS, 00146 Rome, Italy; lucialisa.petrilli@opbg.net; 4 Institute of Transfusion Medicine and Immunology, Medical Faculty Mannheim, Heidelberg University, German Fred Cross Blood Donor Service Baden-Württemberg – Hessen, 68167 Mannheim, GermanyKaren.bieback@medma.uni-heidelberg.de (K.B.); 5 Department of Pathology and Immunology, Medical Faculty, Centre Medical Universitaire (CMU), University of Geneva, 1206 Geneva, Switzerland; beat.imhof@unige.ch

**Keywords:** JAM-C, endothelial progenitors, adhesion, migration, tumor angiogenesis, perivascular niche

## Abstract

The homing of Endothelial Progenitor Cells (EPCs) to tumor angiogenic sites has been described as a multistep process, involving adhesion, migration, incorporation and sprouting, for which the underlying molecular and cellular mechanisms are yet to be fully defined. Here, we studied the expression of Junctional Adhesion Molecule-C (JAM-C) by EPCs and its role in EPC homing to tumor angiogenic vessels. For this, we used mouse embryonic-Endothelial Progenitor Cells (e-EPCs), intravital multi-fluorescence microscopy techniques and the dorsal skin-fold chamber model. JAM-C was found to be expressed by e-EPCs and endothelial cells. Blocking JAM-C did not affect adhesion of e-EPCs to endothelial monolayers in vitro but, interestingly, it did reduce their adhesion to tumor endothelium in vivo. The most striking effect of JAM-C blocking was on tube formation on matrigel in vitro and the incorporation and sprouting of e-EPCs to tumor endothelium in vivo. Our results demonstrate that JAM-C mediates e-EPC recruitment to tumor angiogenic sites, i.e., coordinated homing of EPCs to the perivascular niche, where they cluster and interact with tumor blood vessels. This suggests that JAM-C plays a critical role in the process of vascular assembly and may represent a potential therapeutic target to control tumor angiogenesis.

## 1. Introduction

Over the last two decades, it became clear that tumor vascularization occurs through two distinct but complementary processes, namely, neoangiogenesis (the sprouting and remodeling of pre-existing blood vessels) and vasculogenesis (the de novo formation of blood vessels through the mobilization and recruitment of Endothelial Progenitor Cells (EPCs) into the developing vasculature) [1,2]. Since the first identification of EPCs in adults [3], a substantial number of studies reported an active role of EPCs in postnatal neovascularization [4,5,6,7,8]. However, the relative contribution of EPCs to adult neovasculature is highly debated and has been controversially reported to range from minor [9,10] to relatively high [11]. Using mouse embryonic-Endothelial Progenitor Cells (e-EPCs) [7], we previously demonstrated that their recruitment during tumor angiogenesis in vivo is a multistep process that requires a coordinated sequence of events, similar to leukocyte recruitment to inflammatory tissues [8]. The process comprises the activation of the tumor endothelium, arrest and firm adhesion to the endothelium, transendothelial extravasation into the interstitial space, perivascular formation of cellular clusters and finally interaction of e-EPCs with the angiogenic tumor vasculature [8]. In particular, we demonstrated that E- and P-selectin expressed on tumor endothelium exclusively mediate the initial e-EPC arrest at tumor angiogenic sites [8]. Similar findings were recapitulated using human cord blood-derived EPCs [12].

Members of the Junctional Adhesion Molecule family (JAMs) have been implicated in leukocyte transendothelial migration [13,14,15] and angiogenesis [16]. JAM-A, JAM-B and JAM-C, the three classical members of the JAM family, are characterized by an extracellular region with two immunoglobulin-like domains, a single transmembrane domain and a short cytoplasmic tail with a PDZ-domain-binding motif [17,18]. JAM-C is expressed at vascular cell–cell contacts [19] and was also identified on circulating human platelets, natural killer cells, dendritic cells, B lymphocytes and a subset of T lymphocytes, but not on circulating mouse leukocytes [20,21,22]. Although JAM-C was detected in epithelial cell desmosomes [23], its association with the PDZ proteins ZO-1 and PAR3 suggests a preferential localization in endothelial cell tight junctions [19,24]. JAM-C, like the other members of the JAM family, has been described as potentially capable of forming homophilic (endothelial JAM-C/endothelial JAM-B, endothelial JAM-B/leukocyte JAM-C, endothelial JAM-C/leukocyte ß2 integrins or platelets JAM-C/leukocytes JAM-C) and heterophilic interactions (endothelial JAM-C/endothelial JAM-B, endothelial JAM-B/leukocyte JAM-C, endothelial JAM-C/leukocyte ß2 integrins or platelet JAM-C/leukocyte JAM-C) [17,25,26].

In particular, we and others demonstrated that JAM-C is involved in leukocyte recruitment by mediating transendothelial and reverse transmigration [13,25,27,28]. Moreover, JAM-C was also shown to be involved in angiogenesis and tumor growth [16,29,30,31].

However, to date, no study has investigated the potential role of JAM-C in EPC recruitment during tumor angiogenesis. In this work, we decided to use e-EPCs as a model of endothelial progenitor cells. These cells are well-characterized [32] and represent a more robust model compared to EPCs derived from umbilical cord, for example, which may be subject to higher variability batch-to-batch as a result of differing umbilical cord blood preparations.

We hypothesized that JAM-C, expressed by the tumor endothelium, is responsible for transvascular migration of e-EPCs. In this study, by using noninvasive in vivo multi-fluorescence microscopy techniques, as well as in vitro functional assays mimicking the different steps of EPC recruitment, we addressed this question.

## 2. Results

### 2.1. JAM-C is Expressed by e-EPCs and Localizes at Tight Junctions

**Figure 1:**
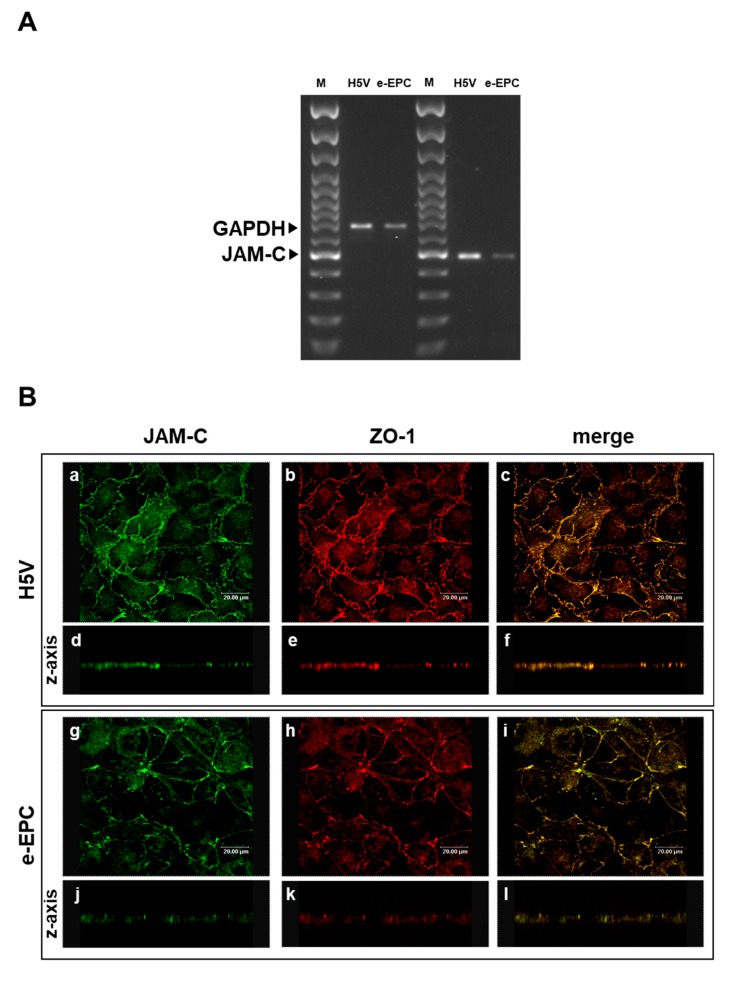
Junctional Adhesion Molecule-C (JAM-C) expression and localization in endothelial cells and embryonal-Endothelial Progenitor Cells (e-EPCs). (**A**) RT-PCR of JAM-C in e-EPCs and in the mouse endothelial H5V cell line (used as positive control). The results shown are representative of 3 independent experiments. GAPDH was used as an internal control. (**B**) Representative images of H5V and e-EPC monolayers showing JAM-C (a, g) and ZO-1 (b, h) expression and distribution at inter-endothelial contacts. Images were merged to analyze the co-localization of JAM-C with ZO-1 (c, i). Images on the z-axis confirmed the co-localization of JAM-C with ZO-1 at tight junctions (d, e, f, j, k, l). Images were acquired using a confocal microscope (Leica TCPSP2 Leica, Bensheim Germany).

As JAM-C is expressed by hemopoietic stem cells and vascular endothelium [33] we hypothesized that JAM-C may also play a role in the recruitment of endothelial progenitor cells at sites of tumor angiogenesis. To address this question, we used mouse embryonic-Endothelial Progenitor Cells (e-EPCs) as a model and analyzed their JAM-C expression. JAM-C mRNA was expressed by e-EPCs, although at slightly lower levels than the H5V endothelial cell line used as positive control (Figure 1A). Confocal microscopy images showed that e-EPCs and H5V cells express JAM-C protein at sites of cell–cell contact (Figure 1B: a, c, g, i). Double staining of JAM-C and ZO-1 showed co-localization of the two proteins at the tight junctions in e-EPC as well as in H5V cells (Figure 1B: b, c, h, i). Images acquired on the z-axis of the cell monolayer confirmed protein localization of JAM-C at the cell junctional regions (Figure 1B: d, e, f, j, k, l).

To test whether e-EPCs and endothelial cells interacted in a homophilic or heterophilic manner [26,34], the expression of JAM-B, a reported counter-receptor of JAM-C, was investigated. Using RT-PCR, we found that JAM-A, but not JAM-B, was expressed by the e-EPCs (Supplementary file 1A). Moreover, we found via FACS analysis that the e-EPCs also did not express β2-integrin, another described receptor of JAM-C (Supplementary file 1B). Thus, based on these findings, we concluded that e-EPCs interact via JAM-C in a homophilic way in trans-orientation.

### 2.2. Role of JAM-C on e-EPC Adhesion and Transendothelial Migration In Vitro

To investigate whether JAM-C mediates e-EPC adhesion to endothelial cells and/or transendothelial migration, we used the anti-JAM-C blocking antibody H33 [30].

**Figure 2:**
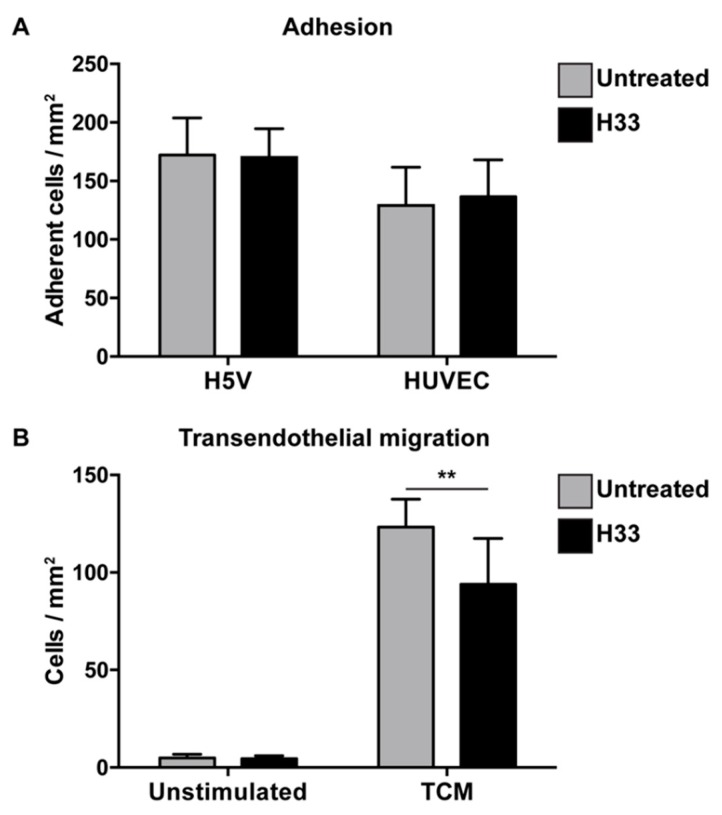
Blocking JAM-C via monoclonal antibody: Effect on e-EPC adhesion and transendothelial migration in vitro. (**A**) Adhesion: Human umbilical vein endothelial cell (HUVEC) monolayers were preincubated in the absence (untreated) or in the presence of H33 anti-JAM-C monoclonal antibody. The fluorescence of cell-tracker green-labeled adherent cells was measured. Data are represented by mean ± SD (*n* = 3). (**B**) Transendothelial migration: A transwell system was used. HUVEC monolayers and e-EPCs were in the absence (untreated) or presence of anti-JAM-C antibody H33. e-EPCs were then plated in the upper chamber onto the HUVEC monolayer to transmigrate in response to tumor-conditioned medium (TCM) or not (unstimulated). Transmigrated cells were stained with DAPI and counted using a fluorescence microscope. Data are represented by mean ± SD (*n* = 3); ** *p* < 0.01.

For adhesion, e-EPCs were allowed to adhere for 20 min on top of confluent endothelial cell (H5V and human umbilical vein endothelial cell (HUVEC)) monolayers in the absence or presence of the anti-JAM-C antibody. The antibody against JAM-C did not affect e-EPC adhesion (Figure 2A).

Based on the strong tumor tropism of e-EPCs in vitro (Supplementary file 2) and in vivo [8], we tested whether JAM-C would be involved in the process of transendothelial migration in response to tumor-conditioned medium. Blockage with H33 anti-JAM-C antibody significantly reduced e-EPC transmigration (Figure 2B).

### 2.3. Inhibition of JAM-C Reduces the Formation of Cord-Like Structures on Matrigel^TM^ In Vitro

**Figure 3:**
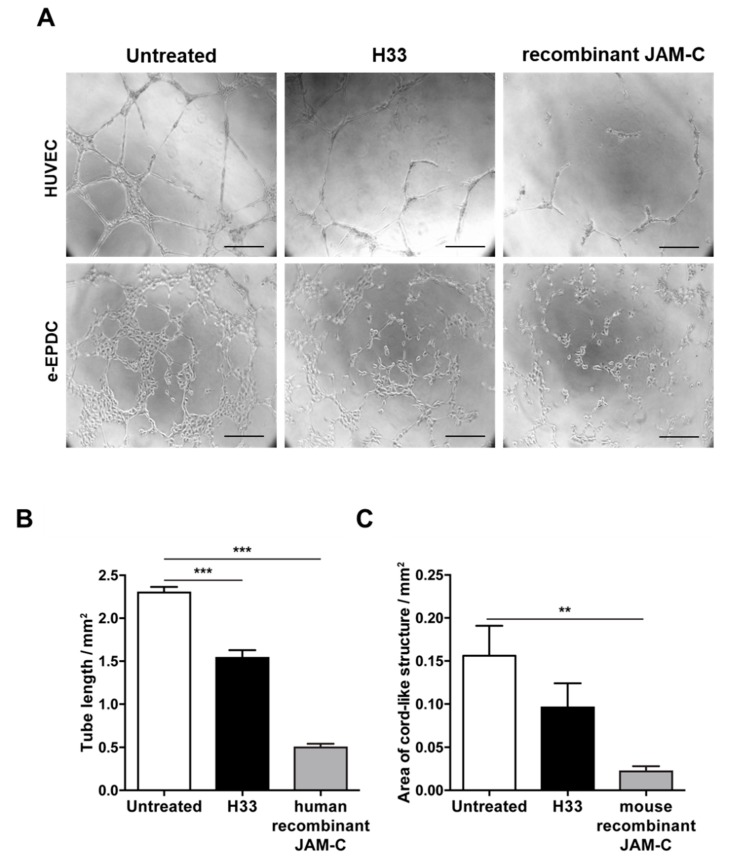
Blocking JAM-C via monoclonal antibody reduces in vitro cord-like structures on Matrigel^TM^. (**A**) Representative images of HUVEC cord-like structures and embryonic-Endothelial Progenitor-Derived Cells (e-EPDCs) cultured on matrigel for 24 h, untreated, treated with anti-JAM-C antibody H33 or with recombinant (r-JAM-C) human JAM-C or mouse JAM-C are shown. (**B-C**) Total tube length was reported for HUVEC (**B**) and the total area of cord-structures for e-EPDCs (**C**). Data are represented by mean ± SD of three separate experiments (** *p* < 0.01, *** *p* < 0.001, compared to control values). Scale bar is 50 μm.

During the complex process of EPC recruitment to tumor blood vessels, important steps include integration into the vascular network and angiogenic sprouting. We have already shown that human adult EPCs are able to be incorporated into the vascular network, both in vitro and in vivo [12,35]. Here, we aimed to understand whether JAM-C contributed to the process. As we previously found, e-EPCs by themselves did not form cord-like structures, but they were able to do so upon treatment with c-AMP, referred to as embryonic-Endothelial Progenitor-Derived Cells (e-EPDCs) [35]. Thus, we performed tube formation assays using e-EPDCs and HUVECs (Figure 3). Inhibition of JAM-C with either anti-JAM-C monoclonal antibody H33 or the soluble recombinant JAM-C (human for HUVECs and mouse for e-EPDCs), significantly reduced the formation of the cord-like structure,= by HUVECs and e-EPDCs (Figure 3A–C; 3B *** *p* < 0.001 and 3C ** *p* < 0.01).

### 2.4. Knockdown of JAM-C Reduces in Vitro Cord-Like Structures on Matrigel^TM^

**Figure 4:**
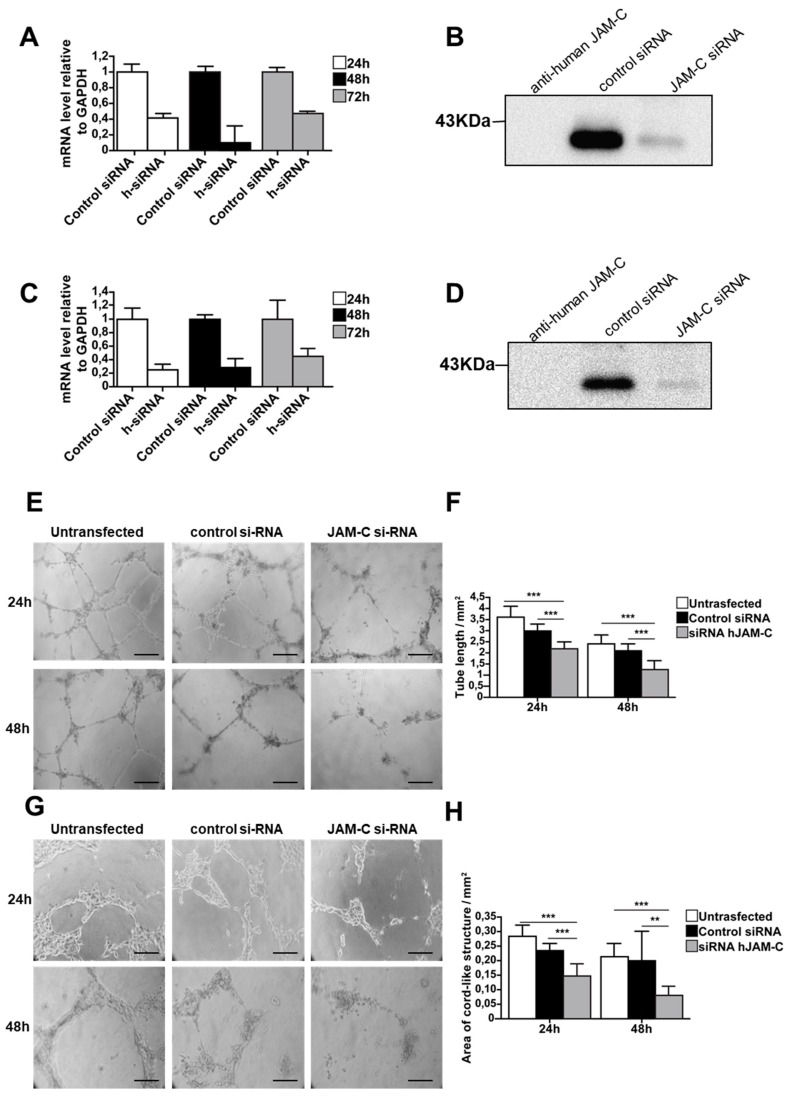
Knockdown of JAM-C reduced in vitro cord-like structures on Matrigel^TM^. (**A**) The mRNA expression levels of human JAM-C in HUVECs transfected with siRNA against JAM-C or with control siRNA isolated at 24, 48 and 72 h after transfection. The 48 h time point showed the strongest knockdown of JAM-C m-RNA levels. Data are represented by mean ± SD (*n* = 3). (**B**) The protein expression level of human JAM-C was analyzed by Western blot in HUVECs transfected with siRNA against JAM-C or with control siRNA, showing a strong decrease in total cellular JAM-C protein levels. Cell surface biotinylation, lysis and JAM-C precipitation were performed 72 h after transfection. Cell lysates from HUVECs transfected with control siRNA or JAM-C siRNA were probed with anti-human JAM-C polyclonal antibody. Equivalent amounts of protein were loaded. As an internal control, anti-human JAM-C was loaded alone. (**C**) The mRNA expression levels of mouse JAM-C showed that JAM-C siRNA treatment decreased JAM-C mRNA levels. (**D**) The protein expression levels of mouse JAM-C showed a strong decrease in total cellular JAM-C protein levels. (**E**) Representative images of cord-like structures from HUVECs untransfected or transfected with control siRNA or with JAM-C siRNA, after 24 h and 48 h on matrigel, are shown. (**F**) Quantitative evaluation of HUVEC cord-like structures, was performed by determining the total tube length. Data are represented by mean ± SD of a representative experiment (*** *p* < 0.001 compared to the untransfected levels and to control siRNA levels, *n* = 12). (**G**) Representative images of cord-like structures from e-EPDC untransfected or transfected with control siRNA or with JAM-C siRNA, after 24 h and 48 h on matrigel, are shown. (**H**) Quantitative evaluation of e-EPDC cord-like structures determining the total area of cord structure. Knockdown of JAM-C significantly reduced the cord-like structure when e-EPDCs were transfected with JAM-C siRNA. Data are represented by mean ± SD of a representative experiment; similar results were observed in separate experiments, with each one performed in triplicate (** *p* < 0.01, *** *p* < 0.001 compared to untransfected levels and to control siRNA levels, *n* = 12). Scale bar is 50 μm.

To further confirm the function of JAM-C during angiogenesis, we used an siRNA approach to directly silence human JAM-C in HUVECs and mouse JAM-C in e-EPCs. Transfection efficiency was tested using control siRNA coupled to Alexa Fluor 488, while MAPK-1 siRNA served as the positive control. Real-time PCR showed that JAM-C siRNA strongly decreased mRNA expression levels of JAM-C in HUVECs and e-EPCs (Figure 4A,C). To check the silencing at the protein level, cells were harvested 72 h after siRNA transfection and JAM-C was immunoprecipitated. Blots showed a strong decrease in JAM-C protein levels with siRNA-treated HUVECs and e-EPCs compared to controls (Figure 4B,C). The JAM-C-silenced cells (HUVECs and e-EPDCs) were then used for the in vitro angiogenesis assay on Matrigel^TM^. Twenty-four hours after seeding, both siRNA-transfected cell types clearly showed thinner tubes compared to control cells (Figure 4E,G). After 48 h, both untransfected and control siRNA cells maintained their cord-like structures, while the tubes almost completely disappeared in the JAM-C siRNA-treated cells. JAM-C-silenced HUVECs and e-EPDCs frequently tended to lose cell–cell contact and remained as single cells. Quantification of total tube length at 24 h and 48 h confirmed a significant decrease in tube formation in both JAM-C siRNA-silenced cell types (Figure 4F, *** *p* < 0.001 and 4H, *** *p* < 0.001, ** *p* < 0.01).

### 2.5. Role of JAM-C on e-EPC Adhesion and Extravasation In Vivo

Our in vitro data suggest that JAM-C mediates recruitment of e-EPCs to the perivascular niche. To investigate the role of JAM-C during e-EPC recruitment to sites of tumor angiogenesis in vivo, we used the syngeneic Lewis-Lung-Carcinoma (LLC) tumor model in the dorsal skin-fold chamber. Between 14 and 17 days after tumor cell implantation, intravital microscopy was performed to visualize the LLC tumor microvasculature. This showed the typical angio-architecture of the LLC tumor model characterized by high angiogenic activity (Supplementary file 3A). JAM-C expression by LLC tumor vessels was examined by fluorescent immunostaining. Co-staining with the endothelial cell marker PECAM-1 (Supplementary file 3B) revealed endothelial-specific staining for JAM-C on LLC tumor vessels, while tumor cells were negative for JAM-C.

**Figure 5:**
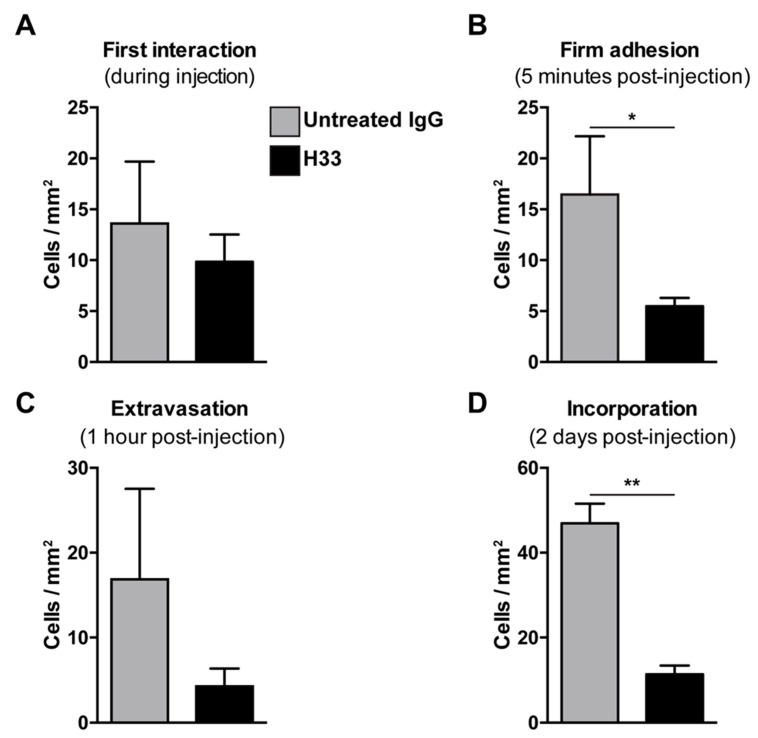
Blocking JAM-C via monoclonal antibody: Effect of e-EPC homing to tumor angiogenesis in vivo. C57BL/6 mice bearing LLC tumors in dorsal skin-fold chambers were pre-injected with Isotype control antibody 9B5 (*n* = 2) or anti JAM-C H33 (*n* = 4). After 20 min, DiI pre-labeled e-EPCs were intra-arterially injected into each group of mice and their recruitment to the tumor microvessels was analyzed by epi-fluorescent intravital video-microscopy. Microscopy and video analysis of cell interactions were performed at different time points: t0 = injection/first interaction (**A**); t1 = 5 min/firm adhesion (**B**); t2 = 1 h/extravasation (**C**), t3 = 2 days/long term recruitment (**D**), showing that JAM-C significantly reduced e-EPCs during firm adhesion and the incorporation steps of e-EPC homing. Data are represented by mean ± SD (* *p* < 0.05, ** *p* < 0.01, compared to IgG control).

Mice bearing LLC tumors were injected with anti-JAM-C H33 or an IgG control antibody. After 20 min, DiI pre-labeled e-EPCs were injected into mice and e-EPC/tumor vessel interactions were analyzed by intravital multi-fluorescence video-microscopy at different time points after e-EPC injection: At time 0, the first interaction was established; 5 min post-injection, firm adhesion was established; at 1 h, extravasation was established; and finally at 2 days post-cell injection, e-EPC incorporation into blood vessels was determined. As previously shown, immediately after cell injection, the circulating e-EPCs homed to the tumor vessel endothelium [8]. At this stage, cells either tethered or adhered to the tumor blood vessel wall. We observed that the e-EPCs injected into H33 pre-treated mice showed a similar initial e-EPC/tumor blood vessel interaction compared to control mice (Figure 5A). However, 5 min after cell injection, a significantly lower number of firm adherent e-EPCs to the tumor blood vessel was found in the H33 treated mice versus the IgG control (Figure 5B).

We previously showed that, within 1 h after cell injection, arrested e-EPCs started to extravasate into the tumor [8]. Thus, we checked e-EPC extravasation 1 h after cell injection and found the number of transmigrated cells was reduced in H33 mice versus IgG mice, although this was not significant (Figure 5C).

### 2.6. Anti-JAM-C H33 Monoclonal Antibody Reduces the Recruitment of e-EPCs into the Perivascular Niche and Leads to Loss of e-EPCs in the Tumor Interstitium

**Figure 6:**
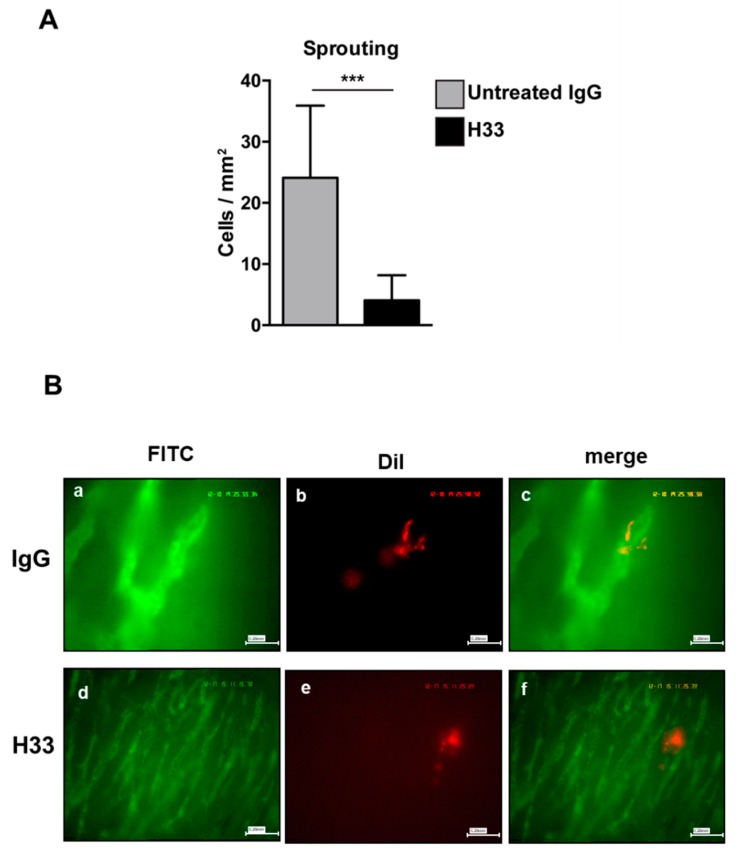
Monoclonal antibody blocking JAM-C affected e-EPC cell morphology in vivo. Mice were treated as described in Figure 5. e-EPC cell morphology (**A**) was analyzed by counting cells showing elongated and polarized shapes. Morphological changes were significantly reduced in the anti-JAM-C H33 group compared with the isotype control antibody 9B5 treated group. Data are represented by mean ± SD (*** *p* < 0.01 H33 vs. IgG; IgG: *n* = 14, H33: *n* = 16). (**B**) Representative images of recruited e-EPC for each group (IgG, H33) on day 2 after cell injection. Tumor microvasculature after contrast enhancement by FITC-dextran (Fluorescein isothiocyanate-dextran) (a, d); DiI-labeled e-EPCs in the same regions of interest (b, e); merged images (c, f) showing e-EPCs characterized mostly by a rounded shape in the H33 group, in comparison with cells recruited in the control IgG-treated mice, which were characterized by an elongated shape.

Next, we studied the integration of e-EPCs into the perivascular tissue. Mice were intraperitoneally (IP) injected daily with H33 anti-JAM-C antibody or control IgG antibody. On day 2 after cell injection, intravital multi-fluorescent video-microscopy was performed and showed a significantly lower number of incorporated e-EPCs in H33 treated mice compared to IgG control mice (Figure 5D). Within a few days after integration, extravasated e-EPCs started to elongate and form clusters in proximity to tumor vascular sprouts [8]. Interestingly, we found that H33-treated mice revealed significantly fewer elongated, clustered cells localized to the perivascular niche compared to the IgG-treated control mice (Figure 6A; ** *p* < 0.01 vs. IgG; IgG: *n* = 14, H33: *n* = 16). The representative intravital video-microscopic images shown in Figure 6B demonstrate how extravasated e-EPCs in the control group were recruited to the perivascular niche, where they clustered and interacted with tumor blood vessels and displayed a clear elongated shape. In contrast, in the H33-treated group, extravasated cells failed to localize to the perivascular niche, retained a rounded shape and did not interact with sprouting tumor blood vessels. They were virtually lost into the interstitial space.

## 3. Discussion

Several studies identified JAM-C as one of the molecules involved in the multistep process of leukocyte recruitment to inflammatory tissues, in particular during the transmigration process [20,23,25,28]. The recruitment of Endothelial Progenitor Cells (EPCs) to sites of tumor angiogenesis is also a multistep process, and although JAM-C was demonstrated in several studies to play a role in angiogenesis and tumor growth [18,30,31,36], none of the studies addressed the role of JAM-C in the context of endothelial progenitor cell recruitment to tumor angiogenesis. To address this question, we first investigated JAM-C expression on EPCs, endothelial cells and tumors. Then, we studied the potential function of JAM-C during the different steps of EPC recruitment to tumor angiogenesis.

Relative to JAM-C expression, JAM-C has been described to be localized at various cell–cell contacts, including vascular endothelial tight junctions [19,37,38] and epithelial desmosomes [23] and also human leukocytes, platelets and polarized spermatids [39]. In line with what was partly reported in previous studies, we found that JAM-C is expressed by endothelial cells and EPCs, and that it co-localizes with the tight junction marker ZO-1. In vivo, we detected JAM-C on tumor blood vessel endothelia (in the LLC1 tumor model), but not on the tumor cells themselves. JAM-C can also engage in homophilic or heterophilic interactions [17,25,26]. We found that e-EPCs express JAM-A but not JAM-B (the JAM-C ligand), and e-EPCs do not express the protein members of the ß2-integrin family such as LFA-1 and MAC-1, the latter of which was reported to be a leukocyte JAM-C counter receptor [28]. This suggested that the interaction between e-EPCs and endothelial cells is mediated by JAM-C/JAM-C homophilic interactions.

To investigate at which step JAM-C is involved in EPC recruitment to develop tumor vasculature, we used a specific monoclonal antibody against JAM-C (H33) that was previously proven to inhibit angiogenesis and tumor growth [30].

In vitro, blocking of JAM-C via H33 slightly affected e-EPC adhesion to and transmigration through endothelial monolayers. Remarkably, H33 more efficiently reduced the formation of cord-like structures. The effect on tube formation was further supported by the use of two additional/complimentary approaches to inhibit JAM-C function, namely, the addition of soluble, recombinant JAM-C protein or JAM-C knockdown with siRNA. We showed that in the absence of JAM-C, blockade of JAM-C or competition by the soluble molecule, both endothelial cells and e-EPDCs were unable to align and connect into a vascular network during the in vitro surrogate angiogenesis assay. These results were important, since they identified JAM-C as a key player in the cascade of e-EPC recruitment to angiogenic sites in tumors and, more specifically, suggested that JAM-C was required for endothelial progenitor cells and mature endothelial cells to form cell–cell contacts, cell clusters and subsequent elongation into a vascular network. To better understand these unexpected findings, in vivo studies were performed to monitor e-EPC recruitment under physiological conditions of tumor angiogenesis.

By using the LLC1 tumor model in the dorsal skin-fold chamber, we looked at the recruitment of e-EPCs to tumor angiogenesis and, by interfering with JAM-C function via H33 monoclonal antibody, we observed effects on multiple steps, including firm adhesion, incorporation and sprouting. For the firm adhesion, the results obtained in vivo were in contrast with those obtained in vitro. This could be explained by the presence in vivo of blood shear stress by flow, which itself strongly affects the e-EPC interactions with blood vascular endothelia, both in vitro and in vivo [12].

Our previous in vivo studies showed that extravasated e-EPCs form clusters and localize in proximity to tumor vascular sprouts within the perivascular niche. After a few days, e-EPCs begin to elongate and eventually interact with tumor endothelium [8]. In this study, we found that blocking JAM-C prevented perivascular incorporation of extravasated e-EPCs, as well as their clustering and interactions with tumor blood vessels. Upon blocking of JAM-C, extravasated cells to the interstitial space remained rounded, isolated and passive. This suggested that JAM-C are needed for e-EPC recruitment to the perivascular niche and interactions with the adjacent tumor endothelium, a critical step in the process of vascular assembly and tumor angiogenesis. These in vivo data are indeed in line with our in vitro results.

Our data are also in line with previous studies that presented evidences for a role of JAM family members in angiogenesis [30,40,41]. Of particular relevance is a study that demonstrated a complete loss of lumen and tube formation by mature endothelial cells after blockage of both JAM-B and JAM-C using antibodies, siRNA or dominant negative mutants [40]. However, blocking of JAM-C in EPCs should be carefully considered, as this could have effects in other contexts too, such as physiological processes like during endometrial regrowth, or other pathophysiological processes like cardiac and retinal ischemia.

Although it is widely accepted that EPCs are involved in the process of tumor angiogenesis, their direct contribution to the composition of new blood vessels is still a matter of debate [5,6,8,11,42,43,44]. We provided evidence for the role of JAM-C as a principal actor of EPC participation in tumor angiogenesis. The recruitment of EPCs to the perivascular niche requires the elongation of cells, alignment of adjacent cells and rearrangement of cell–cell contacts. We propose that JAM-C plays a key role in this scenario and our hypothesis is supported by several pieces of evidence that identified JAM-C as a critical molecule in the organization of the tight junctional and cell polarity complexes [24,38,39,45,46]. Of particular relevance is the recent study that showed JAM-C to be a critical molecule for the differentiation of round spermatids into polarized spermatocytes [39].

In conclusion, we suggest that JAM-C is a potential therapeutic target to control tumor angiogenesis, since an antibody-based approach could inhibit the angiogenic functions of both mature endothelial cells and, in particular, as shown here, endothelial progenitors.

## 4. Materials and Methods

### 4.1. Cells and Cell Culture

Human umbilical vein endothelial cells (HUVECs) were used for the in vitro studies. Umbilical cords were obtained from normal pregnancies immediately after delivery [12]. HUVECs were isolated by trypsin treatment of umbilical cord veins and used at passages 1–6. Cells were grown in culture flasks precoated with 0.5% gelatin (Sigma-Aldrich, Steinheim, Germany) at 37 °C in 5% CO_2_ and cultured in Endothelial Cell Growth Medium (Promocell, Heidelberg, Germany).

The murine immortalized endothelial cell line H5V (a kind gift from Prof. Elisabetta Dejana, IFOM, Milano, Italy) and the murine Lewis Lung Carcinoma cell line (LLC1), were grown in high-glucose Dulbecco’s modified Eagles medium supplemented with 2 mM L-glutamine, 1× Na pyruvate, 1× penicillin/streptomycin and 10% Fetal calf serum (FCS) (Sigma-Aldrich, Hamburg, Germany) at 37 °C in 5% CO_2_. The SF188 human glioma cell line was grown in low-glucose Dulbecco’s modified Eagles medium supplemented with 2 mM L-glutamine, 1X Penicillin/Streptomycin and 10% FCS (all from Sigma-Aldrich, Hamburg, Germany).

Isolation and culture of mouse embryonic-Endothelial Progenitor Cells (e-EPCs) and embryonic- Endothelial Progenitor-Derived Cells (e-EPDCs; obtained upon differentiation with cAMP and retinoic acid) were as previously described [8,32].

To derive tumor-conditioned medium (TCM), SF188 cells were harvested and 1.5 x 10^6^ cells were plated in 75 cm^2^ tissue culture flasks in their specific culture conditions for 4 days. Eighteen hours before collecting the medium, a serum starvation was performed. The medium was removed from the tissue culture flask, the cell monolayers were washed twice with basic Migration Assay Medium (MAM) (serum and protein free DMEM, 25 mM HEPES, 1X penicillin, 1X streptomycin and 2 mM L-glutamine) and 10 mL of warm MAM medium (0.5% Bovine Serum Albumine (BSA) in MAM medium) was added to the cells. After 18 h of serum starvation, the supernatant was collected from the tissue culture flask, centrifuged at 1800 rpm for 10 min and passed through a 0.22 μm filter. Aliquots were stored at –80 °C.

For the in vivo experiments, adherent e-EPCs were fluorescently labeled with the lipid soluble dye DiI (1,1’-dioctadecyl-3,3,3’,3’-tetramethylindo-carbacyanine perchlorate) at 30 µg/mL in normal culture medium for 4 h at 37 °C. Cells were washed twice with PBS 1× and after detachment with trypsin, centrifuged and washed three times with Phosphate buffer saline (PBS) 1×. The cells were resuspended in normal culture medium, filtered through a 40 µm cell strainer and counted. Immediately prior to injection, the cells were centrifuged again and resuspended in 0.9% NaCl.

### 4.2. Immunofluorescence Staining

For immunocytochemistry, cell monolayers were fixed for 5 min with ice-cold methanol, washed in PBS containing 0.1% BSA (Sigma-Aldrich, Steinheim, Germany) and blocked with 2% BSA in PBS for 45 min. Cells were double stained for JAM-C and ZOI-1 by incubation for 2 h at room temperature with polyclonal goat anti-mouse JAM-C (R&D, Wiesbaden, Germany) followed by biotinylated anti-goat IgG (Vector Laboratories, Wertheim-Bettingen, Germany), cy2-streptavidin-conjugated antibody (Jackson Immunoresearch Laboratories) and ZO-1 with rabbit anti-mouse ZO-1 (Zymed Lab, San Francisco, CA, USA), followed by goat anti-rabbit IgG cy3-conjugated (Jackson Immunoresearch laboratories). Images were acquired using a confocal microscope (Leica TCPSP2 Leica, Bensheim Germany).

For immunohistochemistry, 10 µm frozen sections of LLC1 tumor explanted from the dorsal skin fold chamber (*n* = 3; 17 days after the implantation) were fixed for 5 min with ice-cold methanol and dried and rehydrated in PBS, 0.2% gelatin and 0.05% Tween 20 (PGT). Sections were triple stained for 2 h at room temperature for PECAM-1 (Platelet Endothelial Cell Adhesion Molecule-1) as the internal control, with rat monoclonal anti-mouse PECAM-1 (BD, Pharmingen, San Diego, CA, USA), followed by cy3-conjugated anti-rat IgG (Chemicon, Temecula, CA USA), and for JAM-C by incubation with rabbit polyclonal anti-mouse JAM-C followed by FITC-conjugated anti-rabbit IgG (ICN Aurora OH USA) and nuclei stained by DAPI in the mounting medium. Images were acquired using a fluorescent microscope (Axioplan 2 imaging, Zeiss, Jena, Germany) with 400× magnification.

### 4.3. siRNA and Transfection

For transfections, the following reagents and chemically synthesized siRNAs were used: HiPerfect Transfection reagent, a nonsilencing control Alexa Fluor 488 labeled siRNA (to check transfection efficiency), an All Stars negative control siRNA, a positive control siRNA Mn/Hs _MAPK-1, two siRNAs against human JAM-C: Hs_JAM-3_1 HP siRNA, Hs_JAM3_5 HP siRNA and two siRNAs directed against murine JAM-C: Mm_JAM3_1 HP siRNA, Mm_JAM3_4 HP siRNA (all from Qiagen, Hilden, Germany). Combinations of two different siRNAs were transfected at a concentration of 5 nM to produce JAM-C silencing in HUVECs and e-EPDCs. For transfections, e-EPDCs were grown in appropriate culture medium + 10% FCS. In both cases, cells were used in the experiments 72 h after transfection, as described below.

### 4.4. RT-PCR and Analysis

Reverse transcription polymerase chain reaction (RT-PCR) was used to evaluate the expression of JAM-C mRNA in e-EPCs and H5V endothelial cells, which was used as a positive control. Total RNA was isolated from cells using the Genelute Mammalian Total RNA Kit (Sigma-Aldrich, Steinheim, Germany) and subjected to reverse transcription using poly-dT-Primers and M-MLVRT (Moloney Murine Leukemia Virus Reverse Transcriptase)(-H) (Promega, Mannheim, Germany). Single-stranded cDNA was used for PCR amplification according to standard protocols. The primer sequences were as follows (Target, Sequence 5′–3′, Annealing Temperature (°C), MgCl_2_): mouse JAM-C fw: TCG ACA TGG CGC TGA GC, rv: CAG TGT TGC CGT CTT GCC TAC AG, 55 °C, 1.5mM; mouse JAM-A fw: GTA ACT GTA ATG GGC ACC GAG, rv: CAC AGC ATC CAT GTG TGC AGC CTC, 62 °C, 2.5mM; mouse JAM-B fw: CCT GGA CTA TCA TAA GGC AAA TGG, rv: CAT CTT TAA ACC AGA TGT ACT CCG GA, 62 °C, 2.5 mM (RT-PCR for JAM-A and JAM-B, as shown in Supplementary file 1). Control glyceraldehyde 3-phosphate dehydrogenase (GAPDH) amplification was performed under the same conditions as JAM-C PCR and the following primer sequences were used: mouse GAPDH fw: CTC ACT CAA GAT TGT CAG CAA TG, rv: GAG GGA GAT GCT CAG TGT TGG. The PCR products were 460 base pairs (bp) for JAM-C and 668 bp for GAPDH.

Quantitative real-time RT-PCR (qRT-PCR) was performed with the sequence detection system ABI Prism 7700 (Applied Biosystems) using 2× ABgene Master Mix (ABgene, Epson, UK) and the following gene expression assays: h-JAM-C: Hs00230289_m1; m-JAM-C: Mm00499214_m1; h-GAPDH: Hs02758991_g1; m-GAPDH: Mm99999915_g1. GAPDH was used as a housekeeping gene. All reagents were purchased from Applied Biosystems, Darmstadt, Germany.

Levels of gene expression were determined by the comparative CT (threshold cycle) method (DDCT). The relative JAM-C expression was presented as arbitrary units of the ratio of CT of JAM-C over CT of GAPDH.

### 4.5. Cell-Surface Biotinylation/JAM-C Immunoprecipitation

Cell-surface biotinylation was performed with some modifications compared to previously described methods [19,22,28]. HUVEC and e-EPC monolayers were washed three times following incubation with 2 mM NHS-PEO4-biotin (Pierce–Southfield, MI, USA) for 30 min at room temperature. Cell lysates were obtained using 1% Np-40, 50 mM Tris pH 8 and 150 mM NaCl, including protease inhibitors. Approximately 1 μL of rabbit polyclonal anti-human or anti-mouse JAM-C was added to 300 μg HUVEC cell lysate or 250 μg e-EPC cell lysate, respectively, and incubated at 4 °C overnight. Biotinylated JAM-C proteins were precipitated with protein A-Sepharose (Pierce, Rockford, IL, USA), boiled for 10 min in SDS sample buffer and separated using 10% SDS gels. Following transfer to polyvinylidene difluoride membrane, biotinylated proteins were detected using avidin-horseradish peroxidase (1:2000) and enhanced chemiluminescence (Lumiphos WB, Pierce, USA).

### 4.6. In Vitro Cell–Cell Adhesion Assay

The cellular adhesion assay was performed in 96-well plates (Greiner Bio-one) previously coated with 10 µg/mL fibronectin (Sigma-Aldrich, Steinheim, Germany). Confluent HUVEC monolayers were preincubated for 20 min in the absence (control) or the presence of anti-JAM-C antibody H33 [19] and used as matrix for the e-EPC adhesion. e-EPCs were stained with Cell Tracker Green-CMFDA (5-chloromethylfluorescein diacetate) (Molecular Probes, Eugene, Oregon) and after detachment with trypsin were resuspended in RPMI containing 0.5 % BSA. A total of 2 × 10^4^ e-EPCs/well, in the absence (control) or presence of anti-JAM-C antibody H33 (30 µg/mL) were added to the HUVEC monolayer and allowed to adhere for 20 min at 37 °C in 5% CO2. Total fluorescence and retained cell fluorescence were measured in triplicate before and after washing with warm RPMI using a fluorescent plate reader (Titertek Multiscan, Labsystems) at 485 nm excitation and 520 nm emission. HUVEC monolayers in the RPMI medium without fluorescent e-EPCs were used as negative controls. Adherent e-EPCs were quantified as previously described [47].

### 4.7. Invasion Assay

An invasion assay was performed using BD BioCoatTM Matrigel^TM^ Invasion Chambers (inserts and companion 24-well plates, 8-μm pore size; BD Bioscience). Inserts stored at -20 °C, were first allowed to come to room temperature. To rehydrate the matrigel, warm MAM was added to the interior of the inserts and bottom wells (500 μL/insert and bottom chamber) and the plates were incubated for 2 h at 37 °C. The cells were harvested, and cell suspensions of 2 × 10^5^ cells/mL were prepared in MAM. After removing the medium used for the rehydration of the matrigel, 500 μL of cell suspension was placed in each insert and 750 μL of MAM (control) or of tumor conditioned medium (TCM) was placed in the bottom chambers of the transwell system. The plates were incubated for 24 h at 37 °C in 5% CO_2_. The cells that did not invade were removed from the inserts using cotton swabs. Cells that invaded and attached to the underside of the filter were stained with Diff-Quik staining-kit (Allegiance), which contained a fixative and two staining solutions. Each insert was sequentially transferred in each solution and two beakers of water, then allowed to air dry. Once dried, the filters were removed from each insert and placed bottom-side down on a drop of immersion oil on a slide. A second, very small drop of oil was placed on the filter and a glass cover slip was placed on top of that.

### 4.8. In Vitro Transendothelial Migration assay

Transendothelial migration assays were performed in transwells of 6.5 mm filters and 8 µm pores (Corning, Costar, Amsterdam, The Netherlands). Inserts were coated with 10 µg/mL fibronectin from human plasma (Sigma-Aldrich, Steinheim, Germany). HUVECs were seeded on the transwell filters and grown without medium in the lower compartments for 48 h in a humidified atmosphere (37 °C and 5% CO_2_). In parallel wells, before the assay, the confluence of the HUVEC monolayers was verified by staining with 0.1% crystal violet for 30 min followed by two washes with PBS 1×. The monolayers were observed using an inverted microscope. At the beginning of the experiment, 600 µL of migration assay medium (MAM) (serum-free DMEM with 0.5% BSA, 25 mM HEPES, 1× penicillin/streptomycin, and 2 mM L-glutamine) or 600 µL of tumor-conditioned medium (TCM) was added to the lower compartment of the transwell plate. Where indicated, HUVEC monolayers and e-EPCs were preincubated (at 37 °C, 5% CO2) for 20–30 min with 30 µg/mL H33 (anti-JAM-C). In a final volume of 100 µL of MAM, 1 × 10^5^ e-EPCs were added to the upper chambers. To normalize the migration of the progenitor cells to the migration of the endothelial cells in the monolayer, the same experiment was run in parallel without e-EPCs but with MAM in the upper chambers. The experiment was terminated after 20–22 h. Nonmigrated cells were removed from the upper surface using cotton swabs. Cells that migrated to the underside of the filter were fixed in cold methanol for 5 min, washed in ddH2O and the filter was mounted on a glass slide with Mowiol-DAPI. Each experiment was performed in triplicate and the cells were counted on a fluorescent microscope (Axioplan 2 imaging, Zeiss, Jena, Germany) in six fields on one diagonal of each filter.

### 4.9. In Vitro Tube Formation Assay

Matrigel^TM^ (BD Biosciences, Heidelberg, Germany) was added to the wells of a 96-well plate in a volume of 50 µL and allowed to solidify at 37 °C for 30 min. Cells (e-EPDCs: 10 × 104/mL or HUVECs 17 × 104 cells/mL) were added to each well in 100 µL of Endothelial Cell Growth Medium plus supplements. Untransfected cells, control siRNA and JAM-C siRNA cells were used 72 h after siRNA transfection. Where indicated, cells were preincubated with 20 μg/mL r-mouse and r-human JAM-C respectively (R&D Systems) and 60 µg/mL of anti-JAM-C antibody (H33) for 20 min at 37 °C. Cells were then incubated at 37 °C in 5% CO_2_ for 24 h and images were obtained at intervals. Endothelial cord-like structures were quantified using ImageJ software by determining the total tube length from four representative 100× images/well/mm^2^ for HUVECs and the total area of cord structure from four representative 100× or 200× images/well/mm^2^ for e-EPDCs.

### 4.10. Animals

C57BL/6 female mice were used at 12 weeks of age. Experiments were performed in accordance with the approved institutional protocol and the guidelines of the Institutional Animal Care and Use Committee and in conformity with the German Law for Animal Protection and the National Institute of Health Guidelines for Care and Use of Laboratory Animals (Regierungsprasidium Karlsruhe, AZ: 35-9185.81/G-105/04, Karlsruhe, Germany).

### 4.11. LLC1 Tumor in the Dorsal Skin-Fold Chamber Model

We established a syngeneic tumor model by implanting 1 × 10^6^ of highly angiogenic murine Lewis lung carcinoma cells into dorsal skin-fold chambers in C57BL/6 mice (*n* = 3). This transparent chamber model [48] allowed for direct and noninvasive assessment of the tumor microcirculation using intravital microscopy [49]. The tumor mass was identified as previously described [8,50]. Once the tumors established their microvascular system and initiated tumor growth (50 mm^3^), which was by day 17 post-implantation, intravital video-microscopy was used to characterize the tumor vessels by visualizing previous intravenous (IV) injection of fluorescein isothiocyanate (FITC)-Dextran 150,000. Tumors were also explanted from the skin-fold chambers and analyzed for JAM-C expression by immunofluorescence (*n* = 3).

### 4.12. Intravital Fluorescence Videomicroscopy

Intravital multi-fluorescence video-microscopy was performed as previously described [8,49].

On the day of experiment, a polyethylene catheter (PE-10) was inserted into the right common carotid artery for systemic administration of FITC-Dextran and injection of the antibodies or PBS and the e-EPCs [49]. To study the role of JAM-C in the recruitment process of e-EPCs to the tumor endothelium, 20 min prior to e-EPC injection, mice were injected with 100 µg of anti-JAM-C antibody (H33) in 150 µL of PBS 1× (*n* = 4), or 100 µg of anti-human CD44 (9B5, used as irrelevant antibody, rat IgG2a) in 150 µL of PBS 1× (*n* = 2) or 150 µL of PBS 1× (*n* = 5). After visualization of the tumor microvasculature, 3 x 10^5^ DiI-labeled e-EPCs resuspended in 600 μL of physiological saline solution were infused in three 200 μL aliquots (3 injections/animal). This protocol allowed the dynamic interaction between the e-EPCs and the tumor endothelium to be assessed within three different microvascular regions (size: 1.2 mm^2^). During the intra-arterial cell injection, the absolute number of e-EPCs that passed through (100% input) and the number of EPCs which were arrested and adhered to the microvasculature (3 or 4 regions of interest/animal/group) were determined. Adherent e-EPCs were classified as cells that remained attached to the vessel wall for more than 2 s, despite an unaltered microvascular blood flow. To exclude recirculating cells from the analysis, the observation period after cell injection was limited to 20 s and an interval of 3 min was allowed before the next cell infusion [51]. The tumor microvasculature was scanned at 5 min, 1 h and 2 days after cell injection to assess permanent e-EPC–endothelium interactions. The mice were intraperitoneally injected with the same amount of PBS 1×, isotype control antibody or anti-mouse JAM-C for the following two days after cell infusion.

### 4.13. Statistical Analysis

Bar graphs represent the mean ± SD. All experiments were evaluated using two-tailed Student’s t-test or 2-way AVOVA. *p* < 0.05 was considered statistically significant. The symbol * indicated a value of *p* < 0.05, ** *p* < 0.01, *** *p* < 0.001.

## Supplementary Materials

Supplementary Materials can be found at https://www.mdpi.com/1422-0067/21/4/1209/s1.

## Abbreviations

EPCs	Endothelial Progenitor Cells
JAM	Junctional Adhesion Molecule
e-EPDCs	Embryonic-Endothelial Progenitor-Derived Cells
IP	Intraperitoneally
IV	Intravenous

## References

[1] Ding Y.-T., Kumar S., Yu D.-C. (2008). The role of endothelial progenitor cells in tumour vasculogenesis. Pathobiology.

[2] Roncalli J.G., Tongers J., Renault M.-A., Losordo D.W. (2008). Endothelial progenitor cells in regenerative medicine and cancer: A decade of research. Trends Biotechnol..

[3] Asahara T., Murohara T., Sullivan A., Silver M., van der Zee R., Li T., Witzenbichler B., Schatteman G., Isner J.M. (1997). Isolation of putative progenitor endothelial cells for angiogenesis. Science.

[4] Urbich C., Heeschen C., Aicher A., Sasaki K.-I., Bruhl T., Farhadi M.R., Vajkoczy P., Hofmann W.K., Peters C., Pennacchio L.A. (2005). Cathepsin L is required for endothelial progenitor cell-induced neovascularization. Nat. Med..

[5] Lyden D., Hattori K., Dias S., Costa C., Blaikie P., Butros L., Chadburn A., Heissig B., Marks W., Witte L. (2001). Impaired recruitment of bone-marrow-derived endothelial and hematopoietic precursor cells blocks tumor angiogenesis and growth. Nat. Med..

[6] Li B., Sharpe E.E., Maupin A.B., Teleron A.A., Pyle A.L., Carmeliet P., Young P.P. (2006). VEGF and PlGF promote adult vasculogenesis by enhancing EPC recruitment and vessel formation at the site of tumor neovascularization. FASEB J..

[7] Kupatt C., Horstkotte J., Vlastos G.A., Pfosser A., Lebherz C., Semisch M., Thalgott M., Büttner K., Browarzyk C., Mages J. (2005). Embryonic endothelial progenitor cells expressing a broad range of proangiogenic and remodeling factors enhance vascularization and tissue recovery in acute and chronic ischemia. FASEB J..

[8] Vajkoczy P., Blum S., Lamparter M., Mailhammer R., Erber R., Engelhardt B., Vestweber D., Hatzopoulos A.K. (2003). Multistep nature of microvascular recruitment of ex vivo-expanded embryonic endothelial progenitor cells during tumor angiogenesis. J. Exp. Med..

[9] Machein M.R., Renninger S., de Lima-Hahn E., Plate K.H. (2003). Minor contribution of bone marrow-derived endothelial progenitors to the vascularization of murine gliomas. Brain Pathol..

[10] Peters B.A., Diaz L.A., Polyak K., Meszler L., Romans K., Guinan E.C., Antin J.H., Myerson D., Hamilton S.R., Vogelstein B. (2005). Contribution of bone marrow-derived endothelial cells to human tumor vasculature. Nat. Med..

[11] Garcia-Barros M., Paris F., Cordon-Cardo C., Lyden D., Rafii S., Haimovitz-Friedman A., Fuks Z., Kolesnick R. (2003). Tumor response to radiotherapy regulated by endothelial cell apoptosis. Science.

[12] Bieback K., Vinci M., Elvers-Hornung S., Bartol A., Gloe T., Czabanka M., Klüter H., Augustin H., Vajkoczy P. (2013). Recruitment of human cord blood-derived endothelial colony-forming cells to sites of tumor angiogenesis. Cytotherapy.

[13] Corada M., Chimenti S., Cera M.R., Vinci M., Salio M., Fiordaliso F., De Angelis N., Villa A., Bossi M., Staszewsky L.I. (2005). Junctional adhesion molecule-A-deficient polymorphonuclear cells show reduced diapedesis in peritonitis and heart ischemia-reperfusion injury. Proc. Natl. Acad. Sci. USA.

[14] Zen K., Liu Y., McCall I.C., Wu T., Lee W., Babbin B.A., Nusrat A., Parkos C.A. (2005). Neutrophil migration across tight junctions is mediated by adhesive interactions between epithelial coxsackie and adenovirus receptor and a junctional adhesion molecule-like protein on neutrophils. Mol. Biol. Cell.

[15] Wegmann F., Petri B., Khandoga A.G., Moser C., Khandoga A., Volkery S., Li H., Nasdala I., Brandau O., Fässler R. (2006). ESAM supports neutrophil extravasation, activation of Rho, and VEGF-induced vascular permeability. J. Exp. Med..

[16] Cooke V.G., Naik M.U., Naik U.P. (2006). Fibroblast growth factor-2 failed to induce angiogenesis in junctional adhesion molecule-A-deficient mice. Arteriosclerosis, Thrombosis, and Vascular Biology.

[17] Weber C., Fraemohs L., Dejana E. (2007). The role of junctional adhesion molecules in vascular inflammation. Nat. Rev. Immunol..

[18] Garrido-Urbani S., Bradfield P.F., Lee B.P.-L., Imhof B.A. (2008). Vascular and epithelial junctions: A barrier for leucocyte migration. Biochem. Soc. Trans..

[19] Aurrand-Lions M., Duncan L., Ballestrem C., Imhof B.A. (2001). JAM-2, a novel immunoglobulin superfamily molecule, expressed by endothelial and lymphatic cells. J. Biol. Chem..

[20] Johnson-Léger C.A., Aurrand-Lions M., Beltraminelli N., Fasel N., Imhof B.A. (2002). Junctional adhesion molecule-2 (JAM-2) promotes lymphocyte transendothelial migration. Blood.

[21] Liang T.W., Chiu H.H., Gurney A., Sidle A., Tumas D.B., Schow P., Foster J., Klassen T., Dennis K., DeMarco R.A. (2002). Vascular endothelial-junctional adhesion molecule (VE-JAM)/JAM 2 interacts with T, NK, and dendritic cells through JAM 3. J. Immunol..

[22] Santoso S., Sachs U.J.H., Kroll H., Linder M., Ruf A., Preissner K.T., Chavakis T. (2002). The junctional adhesion molecule 3 (JAM-3) on human platelets is a counterreceptor for the leukocyte integrin Mac-1. J. Exp. Med..

[23] Zen K., Babbin B.A., Liu Y., Whelan J.B., Nusrat A., Parkos C.A. (2004). JAM-C is a component of desmosomes and a ligand for CD11b/CD18-mediated neutrophil transepithelial migration. Mol. Biol. Cell.

[24] Ebnet K., Aurrand-Lions M., Kuhn A., Kiefer F., Butz S., Zander K., Meyer zu Brickwedde M.-K., Suzuki A., Imhof B.A., Vestweber D. (2003). The junctional adhesion molecule (JAM) family members JAM-2 and JAM-3 associate with the cell polarity protein PAR-3: A possible role for JAMs in endothelial cell polarity. J. Cell Sci..

[25] Bradfield P.F., Scheiermann C., Nourshargh S., Ody C., Luscinskas F.W., Rainger G.E., Nash G.B., Miljkovic-Licina M., Aurrand-Lions M., Imhof B.A. (2007). JAM-C regulates unidirectional monocyte transendothelial migration in inflammation. Blood.

[26] Keiper T., Al-Fakhri N., Chavakis E., Athanasopoulos A.N., Isermann B., Herzog S., Saffrich R., Hersemeyer K., Bohle R.M., Haendeler J. (2005). The role of junctional adhesion molecule-C (JAM-C) in oxidized LDL-mediated leukocyte recruitment. FASEB J..

[27] Aurrand-Lions M., Lamagna C., Dangerfield J.P., Wang S., Herrera P., Nourshargh S., Imhof B.A. (2005). Junctional adhesion molecule-C regulates the early influx of leukocytes into tissues during inflammation. J. Immunol..

[28] Chavakis T., Keiper T., Matz-Westphal R., Hersemeyer K., Sachs U.J., Nawroth P.P., Preissner K.T., Santoso S. (2004). The junctional adhesion molecule-C promotes neutrophil transendothelial migration in vitro and in vivo. J. Biol. Chem..

[29] Orlova V.V., Economopoulou M., Lupu F., Santoso S., Chavakis T. (2006). Junctional adhesion molecule-C regulates vascular endothelial permeability by modulating VE-cadherin-mediated cell-cell contacts. J. Exp. Med..

[30] Lamagna C., Hodivala-Dilke K.M., Imhof B.A., Aurrand-Lions M. (2005). Antibody against junctional adhesion molecule-C inhibits angiogenesis and tumor growth. Cancer Res..

[31] Rabquer B.J., Amin M.A., Teegala N., Shaheen M.K., Tsou P.-S., Ruth J.H., Lesch C.A., Imhof B.A., Koch A.E. (2010). Junctional adhesion molecule-C is a soluble mediator of angiogenesis. J. Immunol..

[32] Hatzopoulos A.K., Folkman J., Vasile E., Eiselen G.K., Rosenberg R.D. (1998). Isolation and characterization of endothelial progenitor cells from mouse embryos. Development.

[33] Arcangeli M.-L., Bardin F., Frontera V., Bidaut G., Obrados E., Adams R.H., Chabannon C., Aurrand-Lions M. (2014). Function of Jam-B/Jam-C interaction in homing and mobilization of human and mouse hematopoietic stem and progenitor cells. Stem Cells.

[34] Santoso S., Orlova V.V., Song K., Sachs U.J., Andrei-Selmer C.L., Chavakis T. (2005). The homophilic binding of junctional adhesion molecule-C mediates tumor cell-endothelial cell interactions. J. Biol. Chem..

[35] Hecht N., Schneider U.C., Czabanka M., Vinci M., Hatzopoulos A.K., Vajkoczy P., Woitzik J. (2014). Endothelial progenitor cells augment collateralization and hemodynamic rescue in a model of chronic cerebral ischemia. J. Cereb. Blood Flow Metab..

[36] Garrido-Urbani S., Vonlaufen A., Stalin J., De Grandis M., Ropraz P., Jemelin S., Bardin F., Scheib H., Aurrand-Lions M., Imhof B.A. (2018). Junctional adhesion molecule C (JAM-C) dimerization aids cancer cell migration and metastasis. Biochim. Biophys. Acta Mol. Cell Res..

[37] Aurrand-Lions M., Johnson-Leger C., Wong C., Pasquier D.L., Imhof B.A. (2001). Heterogeneity of endothelial junctions is reflected by differential expression and specific subcellular localization of the three JAM family members. Blood.

[38] Satohisa S., Chiba H., Osanai M., Ohno S., Kojima T., Saito T., Sawada N. (2005). Behavior of tight-junction, adherens-junction and cell polarity proteins during HNF-4alpha-induced epithelial polarization. Exp. Cell Res..

[39] Gliki G., Ebnet K., Aurrand-Lions M., Imhof B.A., Adams R.H. (2004). Spermatid differentiation requires the assembly of a cell polarity complex downstream of junctional adhesion molecule-C. Nature.

[40] Sacharidou A., Koh W., Stratman A.N., Mayo A.M., Fisher K.E., Davis G.E. (2010). Endothelial lumen signaling complexes control 3D matrix-specific tubulogenesis through interdependent Cdc42- and MT1-MMP-mediated events. Blood.

[41] Stellos K., Langer H., Gnerlich S., Panagiota V., Paul A., Schönberger T., Ninci E., Menzel D., Mueller I., Bigalke B. (2010). Junctional adhesion molecule A expressed on human CD34+ cells promotes adhesion on vascular wall and differentiation into endothelial progenitor cells. Arterioscler. Thromb. Vasc. Biol..

[42] De Palma M., Venneri M.A., Roca C., Naldini L. (2003). Targeting exogenous genes to tumor angiogenesis by transplantation of genetically modified hematopoietic stem cells. Nat. Med..

[43] Grunewald M., Avraham I., Dor Y., Bachar-Lustig E., Itin A., Jung S., Yung S., Chimenti S., Landsman L., Abramovitch R. (2006). VEGF-induced adult neovascularization: Recruitment, retention, and role of accessory cells. Cell.

[44] Zentilin L., Tafuro S., Zacchigna S., Arsic N., Pattarini L., Sinigaglia M., Giacca M. (2006). Bone marrow mononuclear cells are recruited to the sites of VEGF-induced neovascularization but are not incorporated into the newly formed vessels. Blood.

[45] Scheiermann C., Meda P., Aurrand-Lions M., Madani R., Yiangou Y., Coffey P., Salt T.E., Ducrest-Gay D., Caille D., Howell O. (2007). Expression and function of junctional adhesion molecule-C in myelinated peripheral nerves. Science.

[46] Mandicourt G., Iden S., Ebnet K., Aurrand-Lions M., Imhof B.A. (2007). JAM-C regulates tight junctions and integrin-mediated cell adhesion and migration. J. Biol. Chem..

[47] Chan B.M., Elices M.J., Murphy E., Hemler M.E. (1992). Adhesion to vascular cell adhesion molecule 1 and fibronectin. Comparison of alpha 4 beta 1 (VLA-4) and alpha 4 beta 7 on the human B cell line JY. J. Biol. Chem..

[48] Lehr H.A., Leunig M., Menger M.D., Nolte D., Messmer K. (1993). Dorsal skinfold chamber technique for intravital microscopy in nude mice. Am. J. Pathol..

[49] Vajkoczy P., Ullrich A., Menger M.D. (2000). Intravital fluorescence videomicroscopy to study tumor angiogenesis and microcirculation. Neoplasia.

[50] Vajkoczy P., Schilling L., Ullrich A., Schmiedek P., Menger M.D. (1998). Characterization of angiogenesis and microcirculation of high-grade glioma: An intravital multifluorescence microscopic approach in the athymic nude mouse. J. Cereb. Blood Flow Metab..

[51] Chavakis E., Hain A., Vinci M., Carmona G., Bianchi M.E., Vajkoczy P., Zeiher A.M., Chavakis T., Dimmeler S. (2007). High-mobility group box 1 activates integrin-dependent homing of endothelial progenitor cells. Circ. Res..

