# Supplementary material for: Junctional Adhesion Molecule-C Mediates the Recruitment of Embryonic-Endothelial Progenitor Cells to the Perivascular Niche during Tumor Angiogenesis
Source: Int J Mol Sci. 2020 Feb 11;21(4):1209. doi: 10.3390/ijms21041209 (PMC7072851; doi:10.3390/ijms21041209)
Supplement: Supplementary file 1 [file ijms-21-01209-s001.pdf]

Supplementary material

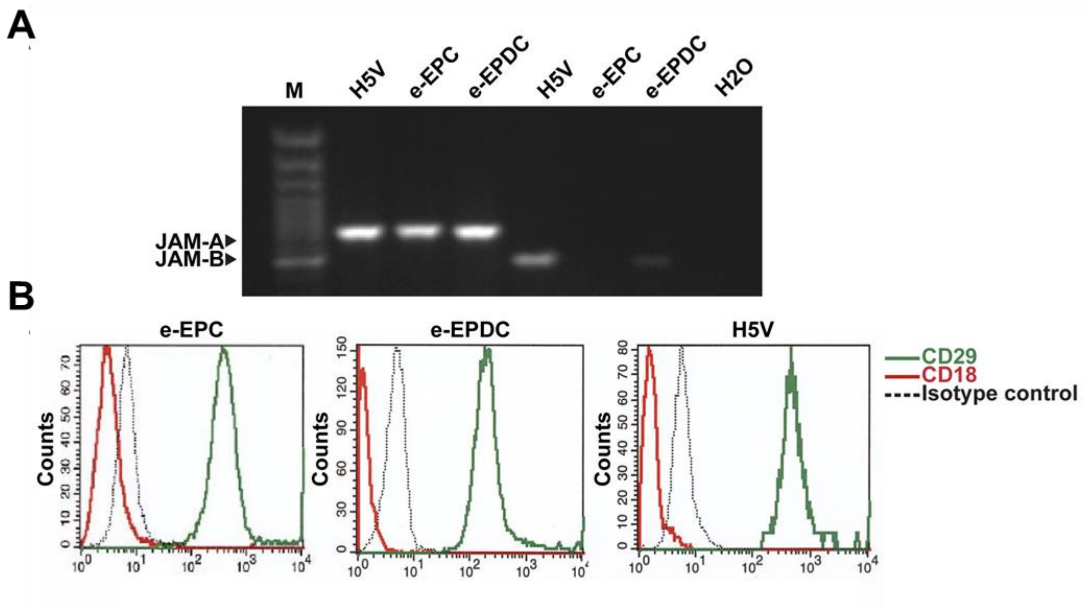

Supplementary Figure 1. e-EPC expression analysis of other JAM-family members and of reported counter receptors of JAM-C. (**A**) RT-PCR expression analysis of JAM-A and JAM-B in e-EPC, e-EPDC and in H5V cell line (used as control). (**B**) FACS analysis of CD18 (β2 integrin) and CD29 (β1 integrin) on e-EPC, e-EPDC and H5V endothelial cells.

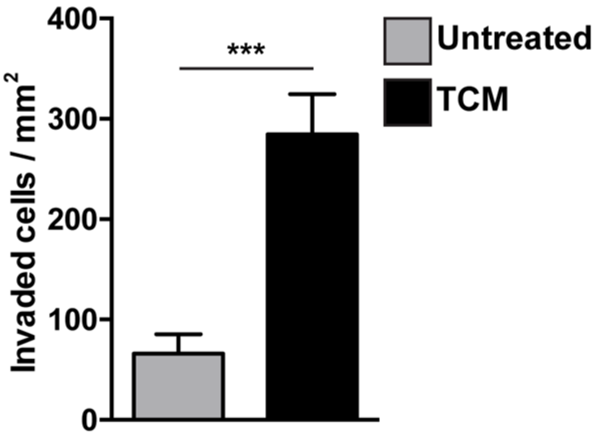

Supplementary Figure 2. e-EPC matrigel invasion assay *in vitro*. Matrigel invasion assay into transwell system of e-EPC in response control medium (MAM) or to tumor conditioned medium (TCM). Data are mean ± SD (n=3); *** $p < 0.001$.

A

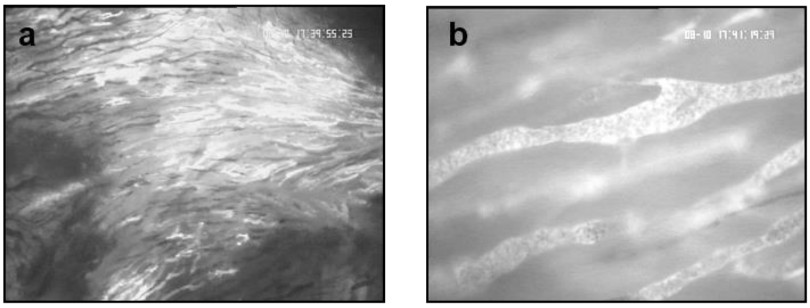

B

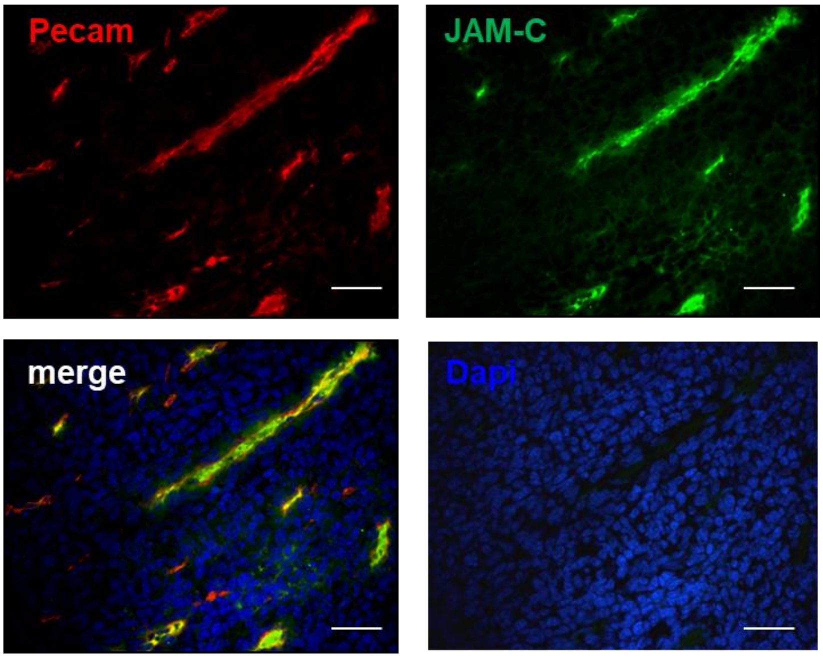

Supplementary Figure 3. Lewis Lung Carcinoma (LLC) tumor in the dorsal skin fold chamber and immunofluorescence analysis of JAM-C on tumor microvessels. (**A**) Representative images of LLC microvasculature on day 17 after tumor cell implantation into female C57BL/6 mice. Note the characteristic parallel pattern of the LLC micro-vessels (a, magnification 5x; b and c magnification 20x). Visualization by intravital epi-fluorescent video-microscopy. Contrast enhancement by fluorescein isothiocyanate (FITC)-Dextran 150,000 i.v. (**B**) Frozen sections of LLC tumor implanted in the dorsal skin fold chamber (n=3; 17 days after implantation) were stained for PECAM-1(red) used as internal control and JAM-C (green). DAPI was used for nuclei staining. Images were merged to confirm the co-localization of JAM-C with PECAM-1 in the tumor microvessels. Images were acquired using a fluorescent microscope (Axioplan 2 imaging, Zeiss, Jena, Germany). Scale bar 20μm.
